# A case of synchronous intramucosal gastric carcinoma with multiple lymph node metastases

**DOI:** 10.1186/s40792-021-01149-z

**Published:** 2021-04-02

**Authors:** En Amada, Hirofumi Kawakubo, Satoru Matsuda, Shuhei Mayanagi, Rieko Nakamura, Tomoyuki Irino, Norihito Wada, Shuji Mikami, Yuko Kitagawa

**Affiliations:** 1 Department of Surgery, Keio University School of Medicine, 35 Shinanomachi, Shinjuku, Tokyo, Japan; 2 Division of Diagnostic Pathology, Keio University School of Medicine, 35 Shinanomachi, Shinjuku, Tokyo, Japan

**Keywords:** Stomach neoplasms, Multiple primary neoplasms, Lymphatic metastasis

## Abstract

**Background:**

In Japan, the prevalence of synchronous multiple intramucosal gastric carcinoma is reported to be 5–15%. Here is a case of a synchronous small gastric carcinoma fulfilling the definite indication and curative criteria for endoscopic submucosal dissection with multiple lymph node metastases.

**Case presentation:**

A Japanese woman in her fifties with a history of endoscopic resection for mucosal poorly differentiated adenocarcinoma was evaluated, with the UICC TNM classification stage being cT1aN0M0 cStageIA. She had undergone total gastrectomy with D1 + lymph node dissection. Histopathological examination revealed 16 individual sporadic lesions in the gastric body, with maximum diameter 3 mm and localization in the lamina propria. Twenty-seven nodes were resected, and metastasis of the carcinoma was revealed in 24 nodes.

**Conclusions:**

Undifferentiated intramucosal gastric cancer has a relatively high probability of lymph node metastasis; however, synchronous early lesions are often overlooked. Frequent follow-up examinations may increase the detection of multiple gastric cancers.

## Introduction

In Japan, the prevalence of synchronous multiple intramucosal gastric carcinoma is reported to be 5–15%. It relatively frequently occurred in elderly males and in patients with adenoma, atrophic gastritis, or a family history of gastric cancer compared to patients with solitary early gastric cancer [1]. We experienced a case of early gastric carcinoma in middle-aged female that was widely scattered troughout the gastric body, each lesion of which had a definite indication for curative resection of ER based on the Gastric Cancer Treatment Guidelines’ (ver. 4) criteria at that time [2]; this case was concluded to have multiple lymph node metastases.

## Case presentation

**Fig. 1:**
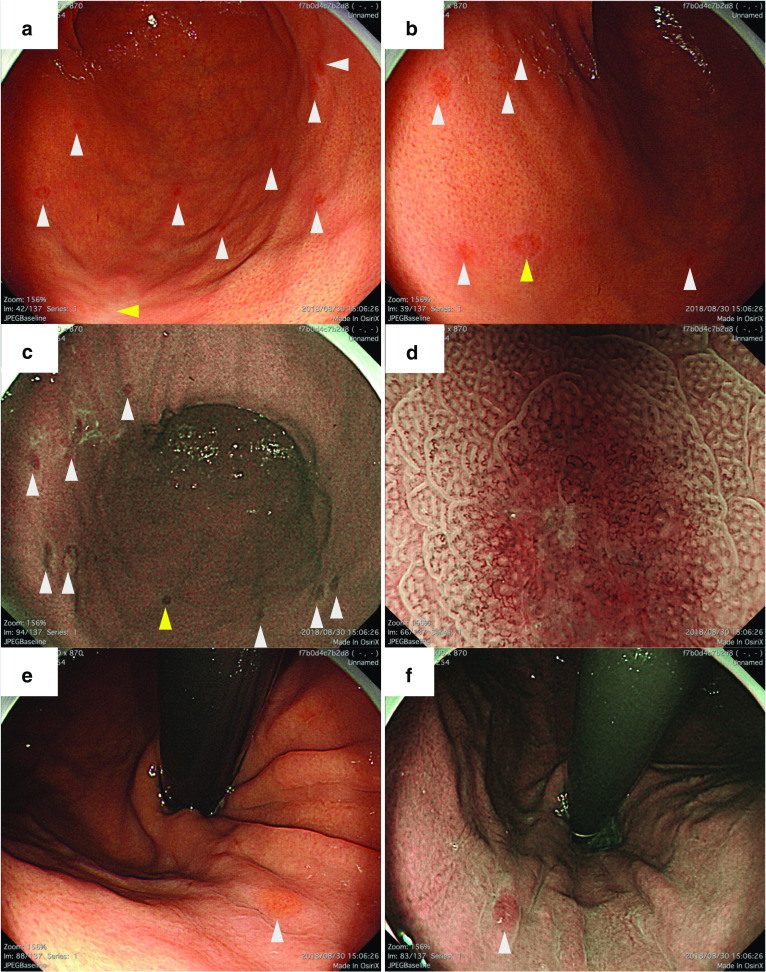
Endoscopic image in the preoperative state. **a** and **b**: White light images of the middle-third and lower-third of the gastric body. More than ten small flat erosive lesions less than 5 mm in diameter were widely scattered (white and yellow arrowheads). By narrow-band imaging (**c**), those lesions were observed as brownish lesions (white and yellow arrowheads). **d** A magnified image of the lesion identified by a yellow arrowhead. A corkscrew-like irregular microvascular pattern with normal grandular structure was observed. **e** and **f**: White light and narrow-band images of lesions located at the upper gastric body

**Fig. 2:**
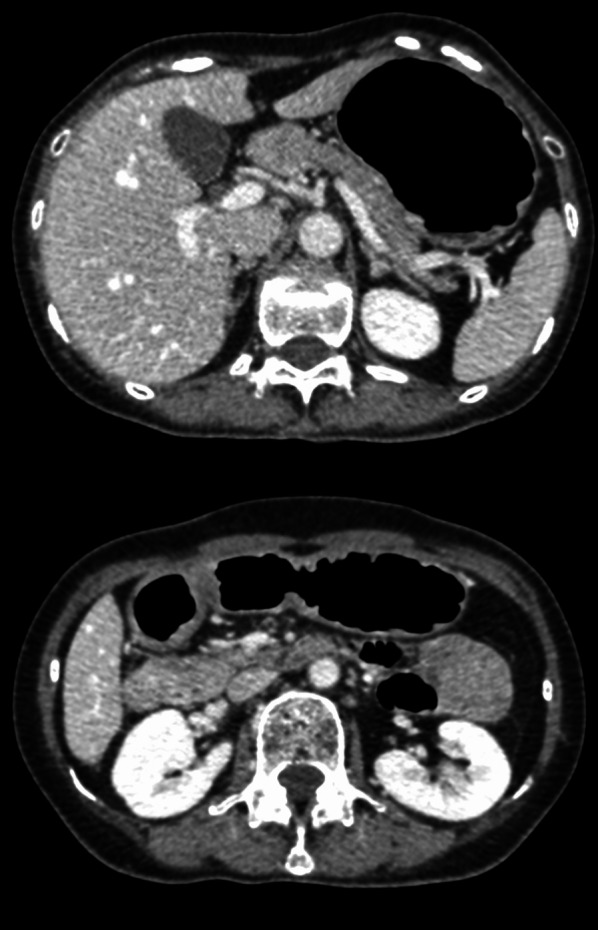
Contrast computed tomography image showed no swelling lymph nodes or distantmetastasis

**Fig. 3:**
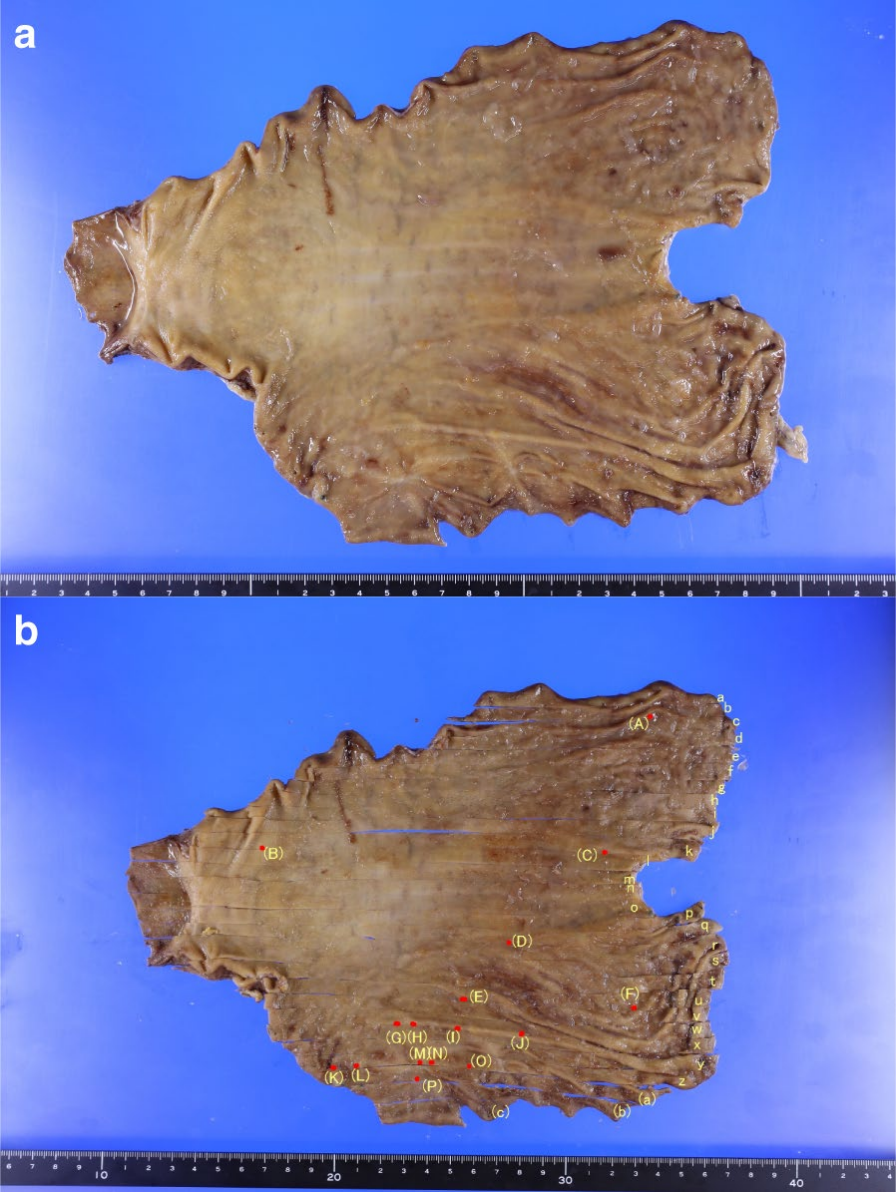
Macroscopic image of the resected specimen. **a** Original formalin-fixed specimen. **b** Mapping of multiple lesions on the gastric body. Sixteen individual sporadic lesions were observed

A Japanese woman in her fifties presented at a clinic with dysphagia. She had no family history of cancer. Upper gastrointestinal endoscope (EGD) had been performed, and two reddened lesions 20 mm in size were observed at the greater curvature of the middle-third and lower-third of the gastric body. A biopsy was taken from one of the lesions and revealed signet-ring cell carcinoma. She was referred to our institution for further treatment. Endoscopic submucosal dissection (ESD) had been performed on these two lesions. The histopathological examination revealed that both lesions were poorly differentiated adenocarcinoma, which was localized in the lamina propria with no lymphatic or vascular invasion. Endoscopists at our institute performed follow-up-EGD performed 4, 7, 8 and 9 months after the ESD, since the initial EGD performed at our institute revealed multiple reddened area with no findings that mentions existence of malignancy at that time. And EGD performed on 9 months after the ESD showed that more than ten small flat erosive lesions less than 5 mm in diameter were widely scattered throughout the middle-third and lower-third of the gastric body (Fig. 1). Magnified narrow-band imaging revealed that all the lesions had a corkscrew-like irregular microvascular pattern with a normal glandular structure, which was presumed to be the existence of poorly differentiated carcinoma cells. A biopsy specimen was obtained from the lesion at the posterior wall of the lower-third of the gastric body. The specimen contained Periodic Acid-Schiff staining positive, pan-cytokeratin (clone: AE1/AE3) positive, and p53 weakly positive cells and was demonstrated to be poorly differentiated adenocarcinoma. Contrasted computed tomography imaging showed no distant metastasis and no swelling of the regional lymph nodes (Fig. 2). Furthermore, Positron Emission Tomography imaging showed no abnormal accumulation of 18F-fluorodeoxyglucose in her body. The physical examination and routine laboratory examination results were unremarkable, including carcinoembryonic antigen (CEA) and carbohydrate antigen (CA) 19-9, which were within normal limits (1.1 ng/ml and 20 U/ml, respectively). Serum Helicobacter-pylori-antibody test and pepsinogen test were negative. The UICC TNM classification staging for her gastric cancer was cT1aN0M0 cStage IA, and each lesion had a definitive indication for ESD; however, the lesions were diffusely located along the entire gastric wall, and ESD was expected to create a large defect of the gastric mucosa. The case was meticulously discussed by a conference of experts, and under full consent of the patient, a total gastrectomy was planned. She underwent robot-assisted total gastrectomy with D1 + lymph node dissection. She was discharged from the hospital on the 8th postoperative day. The final diagnosis was pT1aN3bM0 pStage IIIB, and she began adjuvant chemotherapy with tegafur, gimeracil, oteracil, and oxaliplatin. She is confirmed to be alive with no recurrence at 8 months after the operation (Fig. 3).

## Histopathological findings

Sixteen individual sporadic lesions were observed in the gastric body. The lesions were a maximum of 3 mm in diameter. In each lesion, atypical cells with hyperchromatic nuclei were proliferating in the lamina propria. Each lesion contained Periodic Acid-Schiff staining positive, pan-cytokeratin (clone: AE1/AE3) positive, p53 weakly positive, Chromogranin A negative, and a -fetoprotein negative cells and was demonstrated to be poorly differentiated adenocarcinoma. No lymphatic or vascular invasion was observed. A total of 27 lymph nodes had been resected, and metastasis of the carcinoma was revealed in 24 nodes. The following lymph node stations displayed metastasis of the carcinoma: #1, #3b, #4d, #5, #6, #9, and #11p (the lymph-node station number is based on the Japanese classification of gastric carcinoma: 3rd English edition [3]. The number of resected lymph nodes and metastatic lymph nodes were as follows: #1 1/1, #3 1/1, #4d 1/1, #5 2/3, #6 6/6, #9 5/5, and #11p 2/2 (metastatic/resected), respectively. Molecular biological analysis of resected specimen was tried; however, the amount of tumor tissue was small and it failed.

## Discussion

**Fig. 4:**
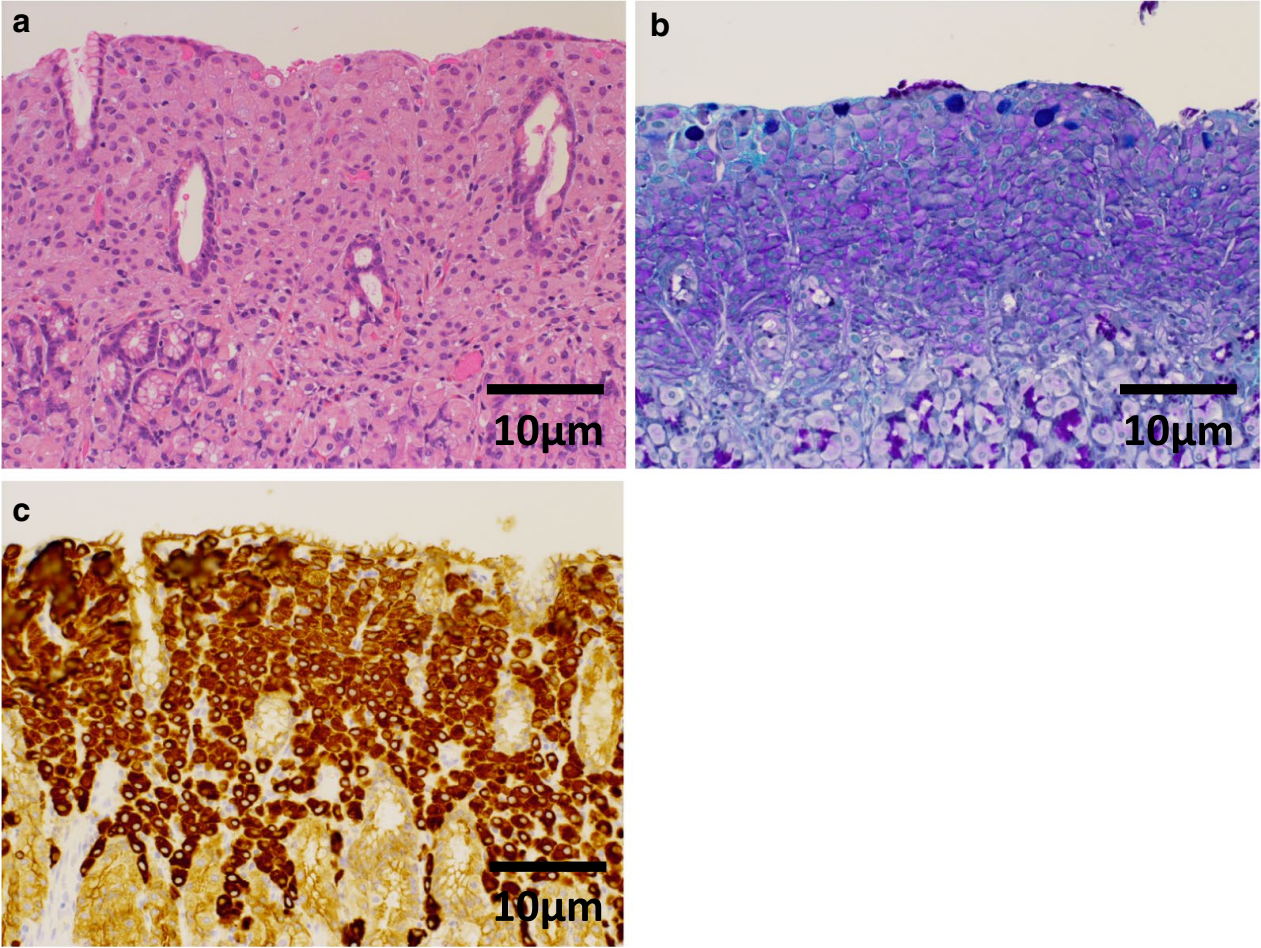
Microscopic image of lesion (O) at Fig. 3. **a** Hematoxylin–eosin (HE) staining, **b** Periodic Acid-Schiff (PAS) and alcian blue staining, and **c** immunostaining of pan-cytokeratin (clone: AE1/AE3). HE staining shows that atypical cells with hyperchromatic nuclei were proliferating in the lamina propria. The lesion contains PAS staining positive, pan-cytokeratin (clone: AE1/AE3) positive cells. No lymphatic or vascular invasion was observed

**Fig. 5:**
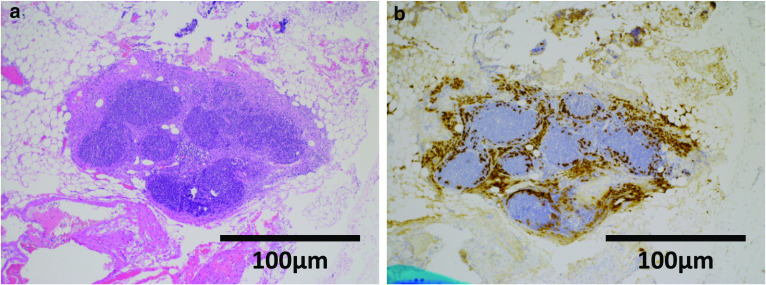
Microscopic image of metastatic lymph node station No. 5. **a** HE staining and **b** immunostaining of pan-cytokeratin (clone: AE1/AE3). Cancer cells are described as pan-cytokeratin (clone: AE1/AE3)

ER has become the standard treatment for intramucosal gastric cancer with virtually no risk of LN metastasis because of its minimal invasiveness. The probability of lymph node metastasis among intramucosal gastric cancer is reported to be 1.2–2.2%. However, undifferentiated intramucosal gastric cancer is reported to have a higher probability of LN metastasis (4.2%) [4]. Seung-Young [5] has reported that undifferentiated-type cancers, including poorly differentiated adenocarcinoma, signet-ring cell carcinoma, poorly cohesive carcinoma, and mucinous adenocarcinoma, are associated with increased risks of LN metastasis (odds ratio 6.104, 95% CI 1.317–28.284, *p* = 0.0021). Among undifferentiated gastric carcinomas, female sex, younger age, larger tumor size (> 20 mm in diameter), the presence of ulcers, and the presence of lymphovascular invasion are considered risk factors for lymph node metastasis [6]. In most reported cases, the metastatic lymph node was localized in the D1 + regional lymph nodes. Furthermore, for synchronous gastric neoplasms, synchronous lesions were reported to have a flat and depressed macroscopic appearance [7]. The absence of Helicobacter pylori infection, lower third location, and the presence of intestinal metaplasia were risk factors for multiple neoplasms [8]. We performed total gastrectomy for synchronous intramucosal gastric cancer all of which fulfilled indication criteria of ER. Seo et al. reported metachronous early gastric malignant lesions had significantly high incidence of containing undifferentiated cancer cells [7]. And all patients who developed metachronous lesions were discovered within 1 year after endoscopic resection. In addition, Fujita et al. reported patients that underwent gastrectomy with combination of the histological differentiation of the main and sublesion of the initial cancers were undifferentiated type had a higher incidence of subsequent remnant gastric cancer [9]. These reports imply that total gastrectomy is supposed to be one option for sporadic early gastric cancer (Figs. 4 and 5).

Existence of specific genetic characteristic is suggested in sporadic intramucosal gastric cancer. Kim et al. reported synchronous gastric neoplasms are correlate with high microsatellite instability (MSI-high) [10]. Patients with MSI-high gastric cancer showed higher prevalence of gastric adenoma than those with microsatellite stable gastric cancers. In addition, MSI-high gastric adenomas, loss-of-functional events of APC is frequently observed, and it is considered to initiates the gastric carcinogenesis and is followed by mutations of histone modifiers and then activation of cancer-related genes [11]. Moreover, Zazula et al. reported gastric cancer with the MSI-high phenotype revealed CDH1 promoter hypermethylation, of which mutation is frequently observed in hereditary diffuse gastric cancer patients [12]. According to The Cancer Genome Atlas project, MSI-high gastric cancer is reported to consist 21.7% of all gastric cancer [13]. In addition, MSI-high gastric cancer showed hypermethylation at the MLH1 promoter, one of the mismatch repair genes. In addition, sporadic signet-ring cell gastric cancer is reported to be one of the key features of gastric cancer triggered by CDH1 mutation [14]. The germline mutation of CDH1 is inherited by autosomal dominant manner and develops Hereditary Diffuse Gastric Cancer (HDGC). The diagnostic criteria of HDGC by International Gastric Cancer Linkage Consortium include: (i) two or more gastric cancer cases in the family, with one being a confirmed diffuse gastric cancer diagnosed before the age of 50 years; (ii) three or more confirmed diffuse gastric cancers in first- or second-degree relatives, independent of age; and (iii) diffuse gastric cancer diagnosed before the age of 40 years without additional family history [15]. In addition, CDH1 testing could be considered in patients with bilateral or familial LBC before the age of 50, patients with DGC and cleft lip/palate, and those with precursor lesions for signet ring cell carcinoma. The clinical feature of this case implies the existence of CDH1 mutation; however, the family history suggests that the mutation might have occurred de novo in this precise case. As for Japanese, only one case of diffuse gastric cancer associated with a de novo genomic deletion of CDH1 gene has been reported so far [16].

## Conclusion

We experienced a synchronous small gastric carcinoma without ulceration and lymphovascular invasion of a relatively young female that emerged after initial ESD. Synchronous lesions are reported to be overlooked in 32.4% of early gastric neoplasia cases [17]; however, the prognosis of small mucosal gastric cancer is favorable, with a reported 5-year relative postoperative survival of 90% or more [18]. As for patients with synchronous early gastric carcinoma, detection frequency of massively invading cancer detected more than in 1 year was reported to be less than 1% [19]. Based on these findings, annual surveillance by EGD is recommended to be performed.

## Data Availability

All data generated or analyzed during this study are included in this published article. We would not like to share data other than those described in the paper, because they include personal information.

## Abbreviations

GCTGs: The Gastric Cancer Treatment Guidelines
ER: Endoscopic resection
EGD: Upper gastrointestinal endoscope
ESD: Endoscopic submucosal dissection
CEA: Carcinoembryonic antigen
CA: Carbohydrate antigen
MSI: Microsatellite instability
APC: Adenomatous polyposis coli
CDH1: E-cadherin
MLH1: Mismatch repair protein
HDGC: Hereditary diffuse gastric cancer
DGC: Diffuse gastric cancers
LBC: Lobular breast cancer
